# ClusterM: a scalable algorithm for computational prediction of conserved protein complexes across multiple protein interaction networks

**DOI:** 10.1186/s12864-020-07010-1

**Published:** 2020-11-18

**Authors:** Yijie Wang, Hyundoo Jeong, Byung-Jun Yoon, Xiaoning Qian

**Affiliations:** 1 School of Informatics, Computing and Engineering, Indiana University, Bloomington, 47405 IN USA; 2 Department of Mechatronics Engineering, Incheon National University, Incheon, 22012 South Korea; 3 Department of Electrical and Computer Engineering, Texas A&M University, College Station, 77843 TX USA; 4 TEES-AgriLife Center for Bioinformatics and Genomic Systems Engineering (CBGSE), Texas A&M University, College Station, 77843 TX USA; 5 Computational Science Initiative, Brookhaven National Lab, Upton, 11973 NY USA

**Keywords:** Comparative network analysis, Multiple network alignment and clustering, Conserved module identification

## Abstract

**Background:**

The current computational methods on identifying conserved protein complexes across multiple Protein-Protein Interaction (PPI) networks suffer from the lack of explicit modeling of the desired topological properties within conserved protein complexes as well as their scalability.

**Results:**

To overcome those issues, we propose a scalable algorithm—ClusterM—for identifying conserved protein complexes across multiple PPI networks through the integration of network topology and protein sequence similarity information. ClusterM overcomes the computational barrier that existed in previous methods, where the complexity escalates exponentially when handling an increasing number of PPI networks; and it is able to detect conserved protein complexes with both topological separability and cohesive protein sequence conservation. On two independent compendiums of PPI networks from *Saccharomyces cerevisiae* (*Sce*, yeast), *Drosophila melanogaster* (*Dme*, fruit fly), *Caenorhabditis elegans* (*Cel*, worm), and *Homo sapiens* (*Hsa*, human), we demonstrate that ClusterM outperforms other state-of-the-art algorithms by a significant margin and is able to identify *de novo* conserved protein complexes across four species that are missed by existing algorithms.

**Conclusions:**

ClusterM can better capture the desired topological property of a typical conserved protein complex, which is densely connected within the complex while being well-separated from the rest of the networks. Furthermore, our experiments have shown that ClusterM is highly scalable and efficient when analyzing multiple PPI networks.

## Background

Advanced high-throughput technologies for measuring protein interactions [1, 2] have provided researchers with rich information about protein-protein interactions (PPI) in various species [3–5]. In order to translate such information into meaningful biological knowledge about the underlying cellular functions and evolutionary mechanisms, one arising computational challenge is how to integrate these PPI data with other available data—such as sequence data—to identify conserved protein complexes that have similar cellular functions across multiple species.

Intuitively, conserved protein complexes should have the following properties with respect to their network topology and sequence homology. Within each species, proteins within a protein complex is densely connected to each other while being loosely connected to and well separated from the rest of the PPI networks [6]. Across different species, conserved protein complexes should contain many orthologs with high sequence similarity. Therefore, it appears reasonable to expect that the problem of identifying such complexes could be effectively addressed by a comparative network analysis approach.

In recent years, efforts have been made to develop computational techniques for the comparison of PPI networks across different species through global network alignment [7–12]. Most of the existing global network alignment algorithms aim to identify the one-to-one mapping with the maximum total similarity of aligned proteins across networks. However, this approach may not directly lead to accurate identification of conserved protein complexes, where an important focus is on grouping proteins that work together towards similar functionalities across species. Furthermore, proteins in conserved protein complexes often have many-to-many orthologous relationships. Local network alignment [13–18] searches for conserved subnetworks across species, which better resembles the task of identifying conserved protein complexes. However, many of them focus on the topological criteria based on conserved edges or other network motifs with specific topological structures, often motivated by conjectured evolutionary or functional models [14, 18, 19]. To the best of our knowledge, none of the existing local alignment algorithms explicitly considers the characteristic topological properties of protein complexes, in which proteins within the complex highly interact with each other but rarely interact with the rest of the network. Therefore, directly applying existing local network alignment algorithms may not result in accurate detection of conserved protein complexes with the maximum coverage of the given PPI networks. Furthermore, both local and global network alignment problems essentially reduce to the (sub)graph isomorphism problem and the alignment results tend to be sensitive to topological errors in the PPI networks. This is certainly problematic, since currently available PPI networks may contain a significant number of false positive interactions while many true interactions are still missing. In comparison, detecting conserved protein complexes is generally more robust to similar topological errors, as we focus on interaction density within complexes and their topological separability (i.e., whether they are well separated from the rest of the PPI networks). In other words, the focus lies on groups of proteins that may potentially belong to the same complex rather than individual proteins, which makes the overall prediction less sensitive to errors in the PPI networks.

In addition to the lack of explicit modeling of the desired topological properties within conserved protein complexes, most of the existing network alignment and clustering algorithms [13–15, 17, 18] do not scale well with the number of species and the network size. For example, it is prohibitive for the network alignment algorithms in [13–15, 17, 18] to handle more than three PPI networks due to the exponential growth of the alignment graph with the number of species and the network size. Finally, to the best of our knowledge, currently, there is neither a gold standard for conserved protein complexes that contains protein complexes from multiple species nor commonly accepted metrics for assessing the performance of algorithms for predicting conserved protein complexes. As a consequence, it has been practically difficult to effectively evaluate the capability of such algorithms to unveil “true” conserved protein complexes, just based on the fact that the identified conserved protein complexes may overlap with well-known protein complexes in another species [13, 14, 17, 18].

To fill these critical gaps in a conserved protein complex identification, we propose a scalable algorithm—ClusterM—that explicitly characterizes the desired topological separability of protein complexes and also incorporates sequence similarity of proteins across the given PPI networks. ClusterM consists of three major steps. The first step is to find a set of protein spines (sets of proteins, one from each network) across different PPI networks. In the second step, well-separated subnetworks around proteins in each protein spine are identified. The final step is to look for conserved subnetworks within those identified well-separated subnetworks, which have both cohesive protein-protein interaction similarity and sequence similarity.

Last but not least, in order to evaluate and compare the performance of ClusterM and other existing algorithms, we have curated a new yeast-human reference conserved protein complex dataset based on yeast and human gold-standard complexes and propose effective evaluation metrics based on the existing measures for assessing protein complex identification algorithms for individual PPI networks. Experimental results based on comparative analysis of yeast and human PPI networks show that ClusterM clearly outperforms the current state-of-the-art algorithms.

## Results

### Identifying conserved complexes in yeast and human networks

We first evaluated ClusterM on the yeast and human PPI networks based on two constructed datasets: DIPYH and IntActYH. In the DIPYH dataset, yeast (*Sce*) and human (*Hsa*) PPI networks are obtained from Database of Interacting Proteins (DIP) version 20150101 [3] and the protein sequence similarity between proteins across networks are computed by BLAST [20]. The human PPI network has 4,278 proteins and 6,446 interactions, and the yeast PPI network has 5,138 proteins and 22,835 interactions. Similarly, the IntActYH dataset contains yeast and human PPI networks extracted from the IntAct database version 20150120 [4] and the corresponding protein sequence similarity across networks. The human PPI network has 23,246 proteins and 106,031 interactions, and the yeast PPI network has 6,392 proteins and 78,287 interactions. We compared ClusterM with the state-of-the-art algorithms—AlignNemo [17], AlignMCL [21], MaWISh [14], NetworkBLAST [22], and NetworkBLAST-M [23]. The selection of parameters of different methods is discussed in the Methods section. We assessed the quality of the identified conserved protein complexes based on the yeast-human reference conserved protein complexes generated from hand-curated yeast and human protein complex data obtained from CYC2008 [24] and the Comprehensive Resource of Mammalian protein complexes (CORUM) [25]. The detailed description of the reference complexes can be found in the Methods section.

In this study, we define a composite score for performance evaluation, which consists of (i) the fraction of the reference conserved protein complexes matched by at least one identified conserved protein complex, (ii) the accuracy score [6], and (iii) the maximum matching ratio for conserved complexes (MMRC). The description of these three evaluation metrics is detailed in the Methods section.

**Fig. 1:**
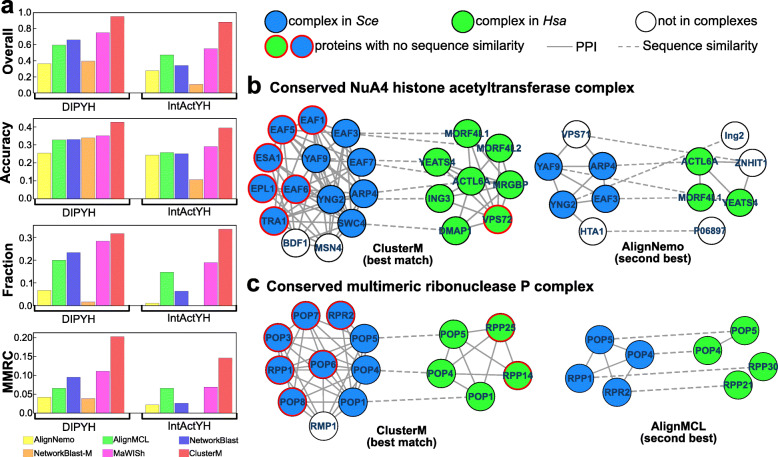
**a** Performance comparison on the yeast-human reference conserved protein complex dataset. NetworkBLAST-M failed to obtain meaningful results due to the large sizes of the PPI networks in IntActYH. **b** The conserved NuA4 histone acetyltransferase protein complex detected by ClusterM and AlignNemo. **c** The conserved multimeric ribonuclease P complex detected by ClusterM and AlignMCL. The nodes with red border edges represent the proteins that do not bear any sequence similarity between two PPI networks. Solid lines denote protein-protein interactions and gray dotted lines indicate sequence similarity

As shown in Fig. 1a, ClusterM outperformed all competing algorithms by a large margin on every evaluation metric for both datasets. Figure 1b and c illustrate two conserved protein complexes identified by ClusterM that cannot be fully recovered by other methods. Clearly, ClusterM utilizes the network topology to facilitate the detection of conserved protein complexes without considering the restriction shared by most of the algorithms (AlignNemo, AlignMCL, NetworkBLAST, and MaWISh), which is only proteins with similar sequences across species can exist in the predicted conserved complexes. NetworkBLAST-M does not have such a restriction but failed to characterize the property of the conserved complexes.

**Fig. 2:**
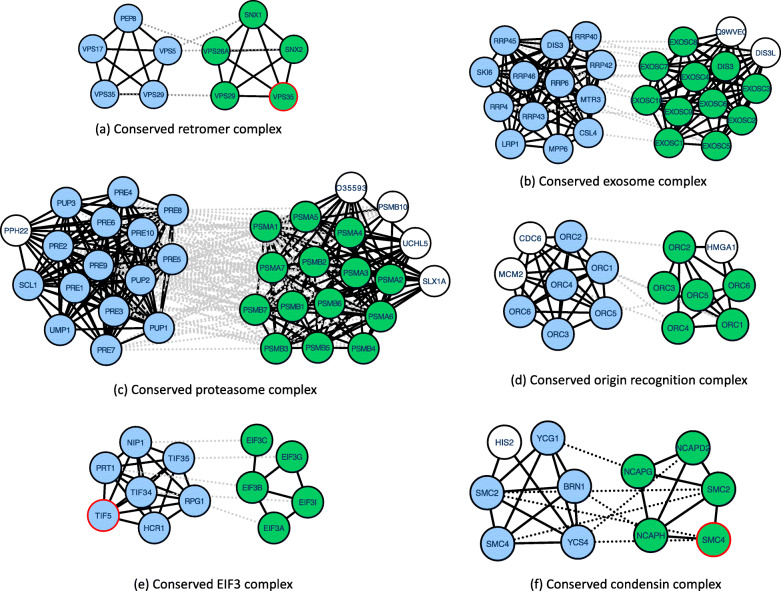
Examples of conserved protein complexes identified by ClusterM on DIPYH and IntActYH. Blue nodes represent proteins in the corresponding reference protein complexes in yeast PPI network. Green nodes represent proteins in the corresponding reference protein complexes in human PPI network. White nodes are proteins that do not belong to the reference protein complexes. Proteins with red bold edges are not identified by ClusterM. Proteins are annotated with their gene names. Solid lines denote protein-protein interactions and dashed lines denote sequence similarity

We discuss two specific conserved protein complexes in Figs. 1b and c in detail to highlight the advantages of ClusterM over other competing methods. In Fig. 1b, there are a relatively larger number of unidentified proteins in the predicted conserved protein complex detected by AlignNemo. The precision of the identified complex is also small as it includes a larger number of irrelevant proteins such as Ing2, P06897, and ZNHIT1 in the conserved protein complex in human PPI network and VPS71 and HTA1 in yeast PPI network. However, ClusterM correctly predicted a larger number of proteins in the NuA4 histone acetyltransferase protein complex with a higher precision. One possible explanation for the significant difference is that ClusterM can correctly identify the proteins without a sequence similarity by effectively utilizing the sequence information and topological structure of PPI networks. On the contrary, AlignNemo failed to identify the proteins in the candidate complexes if they do not have corresponding homology with high sequence similarity. This illustration clearly shows the distinctive advantages of ClusterM over the competing methods to identify conserved protein complex across different PPI networks. In Fig. 1c, AlignMCL also failed to identified the proteins without homology information. Moreover, the predicted conserved complex in human PPI network is ill-connected. That is, only POP4 and POP5 are connected and RPP21 and RPP30 are not connected. This illustration shows that AlignMCL could have a limitation to effective utilization of the topological separability of the conserved protein complexes and AlignMCL can fail to identify the inserted (or deleted) proteins in the conserved complex if there are insufficient or noisy sequence similarity information. In Fig. 2, we show other representative examples of conserved protein complexes detected by ClusterM, which cannot be correctly identified by other algorithms. We have also applied Gene Ontology (GO) enrichment analysis to every identified conserved complexes and the results indicate that ClusterM achieves remarkably higher coverage (i.e., total number of proteins in the predicted complexes) than all competing methods and more than 90% of the predicted complexes identified by ClusterM are significantly enriched in certain GO terms (detailed results and discussions can be found in Supplementary Materials).

### Robustness analysis

To investigate the degree to which the competing algorithms are sensitive to small changes in the input so that meaningful results can be derived even with noisy or incomplete data, we performed a robustness analysis of the algorithms considered in this study. For each algorithm, we optimized the parameters based on the original DIPYH dataset (yeast and human PPI from DIP database and the protein sequence similarity across species) and tested the performance on a perturbed DIPYH dataset, in which we re-wired 10% edges in both yeast and human PPI networks through the M-P procedure [26] to instill topological noise. Furthermore, we treated the protein sequence similarity between yeast and human as a bipartite network, and thereby perturbed 10% of the homologous relationships (*i.e.*, a sequence similarity) also using the M-P procedure [26]. To remove the randomness in the M-P procedure, we generated 10 perturbed networks for each test case and reported the averaged scores.

**Fig. 3:**
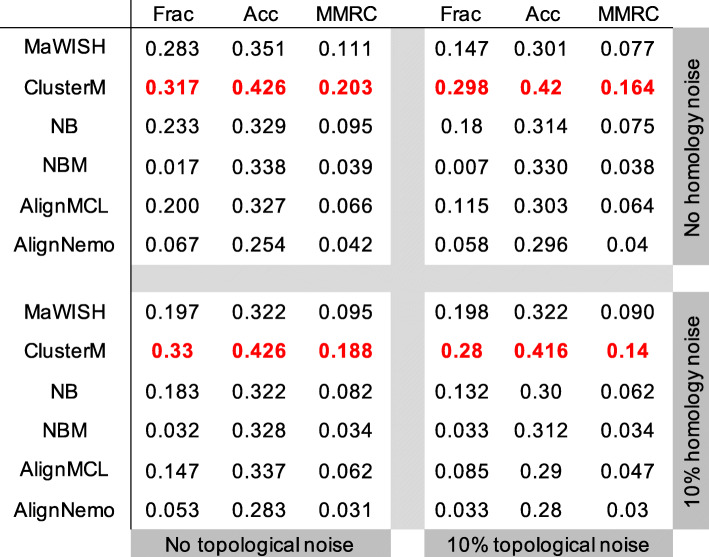
Robustness analysis on yeast and human PPI networks from DIP dataset. We display the fraction, accuracy and MMRC scores for dataset with different noise level. There are four small tables in the figure. The top-left table shows scores for noise free data. The top-right table exhibits scores for data with 10% topological noise but 0% homology noise. The bottom-left table shows scores for 10% homology noise but 0% topological noise. The bottom-right table displays scores for data with 10% topological noise but 10% homology noise

Figure 3 shows the evaluation results—*i.e.*, fraction, accuracy, and MMRC scores—based on four datasets: the noise-free dataset, the dataset with only topological noise, the dataset with only homology noise, and the dataset with both topological and homology noise. Each table corresponds to each dataset in Fig. 3. We first computed the composite score by summing over the fraction, accuracy and MMRC scores. Then, we computed the mean and standard deviation of the four composite scores. The mean of the composite score for ClusterM, MaWISh, NetworkBLAST (NB), AlignMCL, NetworkBLAST-M (NBM), and AlignNemo were 0.890, 0.588, 0.552, 0.486, 0.405, and 0.368, respectively. The standard deviation of the composite score for ClusterM, MaWISh, NB, AlignMCL, NBM, and AlignNemo were 0.066, 0.075, 0.073, 0.079, 0.047, and 0.053, respectively. We can see that ClusterM achieves the largest mean and the third smallest standard deviation among all compared algorithms, which implies that ClusterM is relatively less sensitive to small changes in the input data as well as the parameter selection. Although AlignNemo achieved the smallest standard deviation, it showed the smallest composite score as well and there is a clear gap to the composite score of ClusterM. MaWISh attained the second best composite score for the noise-free dataset, but for noisy datasets, its performance was unstable. This unstable behavior may be due to its dependence on the choice of the seven parameters. Even for relatively small changes in the input, those seven parameters may have to be simultaneously (and significantly) changed to obtain the best performance, which may make it practically difficult to find the optimal parameters for large datasets in a robust manner. NB and NBM showed similar performance in term of the changes of the composite scores, and they turned out to be more sensitive to noise compared to ClusterM but less sensitive when compared to MaWISh. These results show that ClusterM is the most user-friendly algorithm among the compared methods, considering that it is robust to noise and that it does not require the joint optimization of multiple parameters to achieve good performance.

### Conserved protein complex identification for multiple networks

**Fig. 4:**
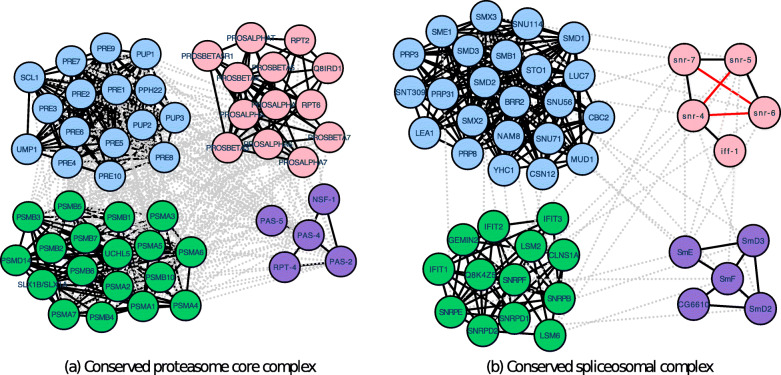
**a** The conserved proteasome core complex identified by ClusterM. Yeast, human, fly, and worm proteins are shown in light blue, green, pink and purple, respectively. Gray dotted lines indicate protein sequence similarity and blue solid lines represent protein-protein interactions. **b** The conserved spliceosomal complex identified by ClusterM. Yeast, human, fly, and worm proteins are shown in light blue, green, pink and purple, respectively. Gray dotted lines indicate protein sequence similarity and blue solid lines represent protein-protein interactions. Red solid lines are PPIs in STRING database

In this section, we present and discuss a specific example that demonstrates the potential of ClusterM in identifying conserved protein complexes in multiple (more than two) PPI networks. We have constructed two PPI datasets for benchmarking protein complex identification across four PPI networks: DIPPPIs and IntActPPIs, based on the corresponding PPI networks and protein amino acids sequences for human (*Hsa*), yeast (*Sce*), fly (*Dme*), and worm (*Cel*) from DIP (version 20150101) and IntAct (version 20150120). The detailed description to construct PPI networks can be found in the Method section. Table 1 provides a basic statistical summary and the abbreviation of PPI networks. In Fig. 4a, we show the conserved proteasome core complex identified by ClusterM in IntActPPIs. This complex was completely missed by all other algorithms that were considered in this study. Proteins in light blue, green, pink, and purple represent proteins in yeast, human, fly, and worm, respectively. We further tested the statistical significance of each of the four protein complexes in the respective organisms based on the GO term annotated to proteasome core complex (GO:0005839). The *p*-values associated to the corresponding yeast, human, fly, and worm complexes are 5.58E-41, 2.80E-48, 4.88E-16, and 5.48E-9, respectively. In Fig. 4b, the spliceosomal complex (GO:0005681) is significantly enriched in the yeast, human, fly, and worm conserved complex with the *p*-values 2.37E-08, 4.16E-12, 6.03E-11, and 2.97E-08, respectively. Note that red solid lines are PPIs missed in IntAct database but we can identify the interactions in STRING database [27]. The enrichment analysis of these conserved protein complexes, which could be detected solely by ClusterM, clearly shows that ClusterM can effectively mine biologically meaningful protein complexes in multiple large-scale PPI networks.

**Table 1:** Abbreviation and information for protein-protein interaction (PPI) networks used in the study

Dataset	Database	Version	Species	Abbreviation	#. proteins	#. interactions
DIPPPIs	DIP	20150101	Yeast	SceDIP	5,138	22,835
	DIP	20150101	Human	HsaDIP	4,278	6,446
	DIP	20150101	Fly	DmeDIP	7,679	23,182
	DIP	20150101	Worm	CelDIP	2,712	4,117
IntActPPIs	IntAct	20150120	Yeast	SceIntAct	6,392	78,287
	IntAct	20150120	Human	HsaIntAct	23,246	106,031
	IntAct	20150120	Fly	DmeIntAct	11,517	41,483
	IntAct	20150120	Worm	CelIntAct	9,721	16,668

### High-level GO term consistency

To examine the biological significance of the conserved protein complexes identified by ClusterM and other existing algorithms, we calculated the coverage and the mean normalized entropy (MNE) score [7] based on high-level GO annotations for the results obtained by ClusterM, MaWISh, AlignMCL, and NetworkBLAST-M. Note that the lower MNE indicates that the proteins in the predicted conserved complex are more consistent in terms of GO annotations. We selected the three algorithms—MaWISh, AlignMCL, and NetworkBLAST-M—as they show the best performance among all compared algorithms except for ClusterM. For the blanks in Tables 2 and 3, MaWISh and AlignMCL cannot deal with more than 3 networks. We applied these four algorithms to identify conserved protein complexes for every combination of PPI networks in DIPPPIs, where the obtained results are presented in Table 2. The evaluation results based on IntActPPIs datasets can be found in Table 3. Both tables reveal that the conserved protein complexes identified by ClusterM consistently achieve lower MNE and higher coverage scores, where it means that ClusterM can predict a larger number of proteins with a high functional consistency.

**Table 2:** GO consistency and coverage comparison on DIPPPIs dataset

PPI networks	measure	MaWISh	AlignMCL	NB-M	CM1	CM10	CM100
SceDIP+HsaDIP	MNE	5.274	3.766	6.110	4.195	4.224	**3.912**
	Coverage	1013	1379	814	**2533**	2406	942
SceDIP+DmeDIP	MNE	3.149	2.434	3.383	2.300	2.286	**2.204**
	Coverage	1327	1945	1242	**3496**	3400	1395
SceDIP+CelDIP	MNE	3.105	2.476	3.490	2.344	2.339	**2.134**
	Coverage	383	809	488	**1140**	1099	353
HsaDIP+DmeDIP	MNE	5.823	4.976	6.252	4.753	4.734	**4.757**
	Coverage	1076	2909	1538	**4239**	3952	904
HsaDIP+CelDIP	MNE	6.972	5.359	6.483	4.937	4.978	**4.724**
	Coverage	260	1202	554	**1656**	1542	349
DmeDIP+CelDIP	MNE	3.366	2.599	3.317	2.246	2.232	**2.123**
	Coverage	522	1706	1044	**1988**	1928	403
SceDIP+HsaDIP+DmeDIP	MNE			6.168	4.470	**4.444**	4.614
	Coverage			1895	**3628**	3410	1525
SceDIP+HsaDIP+CelDIP	MNE			6.425	**4.572**	4.611	4.720
	Coverage			886	**2415**	2232	814
SceDIP+DmeDIP+CelDIP	MNE			3.746	2.554	2.563	**2.414**
	Coverage			1162	**2736**	2659	1200
HasDIP+DmeDIP+CelDIP	MNE			6.616	4.527	4.542	**4.136**
	Coverage			1610	**2762**	2516	697
SceDIP+HsaDIP+DmeDIP+CelDIP	MNE			5.981	4.305	4.339	**4.149**
	Coverage			1351	**2049**	1871	599

Abbreviations: CM1 = ClusterM(*λ*=1), CM10 = ClusterM(*λ*=10), CM100 = ClusterM(*λ*=100), NME = Mean Normalized Entropy, NB-M = NetworkBLAST-M

Bold values denote the best scores corresponding to specific criteria

**Table 3:** GO consistency and coverage comparison on IntAct dataset

PPI networks	measure	MaWISh	AlignMCL	NB-M	CM1	CM10	CM100
SceIntAct+HsaIntAct	MNE	4.734	3.137	4.028	3.089	3.103	**3.018**
	Coverage	1552	4181	478	**6355**	6216	3023
SceIntAct+DmeIntAct	MNE	3.679	2.705	4.208	**2.307**	2.310	2.446
	Coverage	1548	1290	423	**2403**	2391	1209
SceIntAct+CelIntAct	MNE	3.378	2.918	3.679	2.647	**2.635**	2.697
	Coverage	797	818	346	**1578**	1543	637
HsaIntAct+DmeIntAct	MNE	7.587	4.148	4.988	**3.421**	3.477	3.668
	Coverage	4413	5898	219	**7069**	6730	1941
HsaIntAct+CelIntAct	MNE	6.463	4.490	4.639	3.449	3.448	**3.367**
	Coverage	2263	3724	167	**4077**	3907	1202
DmeIntAct+CelIntAct	MNE	4.497	4.013	5.154	2.745	2.785	**2.477**
	Coverage	545	1120	204	**1891**	1833	520
SceIntAct+HsaIntAct+DmeIntAct	MNE			4.317	**3.154**	3.165	3.315
	Coverage			631	**8640**	8354	4233
SceIntAct+HsaIntAct+CelIntAct	MNE			3.848	**3.294**	3.307	3.352
	Coverage			408	**6160**	5952	2675
SceIntAct+DmeIntAct+CelIntAct	MNE			4.479	2.657	2.659	**2.574**
	Coverage			640	**3321**	3222	1282
HasIntAct+DmeIntAct+CelIntAct	MNE			5.321	3.196	3.246	**3.109**
	Coverage			435	**6213**	5895	2168
SceINTACT+HsaINTACT+DmeINTACT+CelINTACT	MNE			4.344	**3.139**	3.153	3.252
	Coverage			456	**5907**	5668	2246

Abbreviations: CM1 = ClusterM(*λ*=1), CM10 = ClusterM(*λ*=10), CM100 = ClusterM(*λ*=100), NME = Mean Normalized Entropy, NB-M = NetworkBLAST-M

Note: Bold values denote the best scores corresponding to specific criteria

## Discussion

In this paper, we proposed a scalable algorithm—ClusterM—that can identify conserved protein complexes by integrating protein sequence information and topological structure of the PPI networks. As demonstrated by our results, ClusterM can better capture the desired topological property of a typical conserved protein complex, which is densely connected within the complex while being well-separated from the rest. Experimental results based on real-world PPI networks and protein complexes show that ClusterM significantly outperforms other state-of-the-art algorithms in the task of identifying conserved protein complexes. Additionally, the conserved protein complexes identified by ClusterM have been shown to boast better high-level GO term consistency compared to other competing algorithms.

ClusterM is an enhanced approach that directly considers the characteristic topological structure of conserved protein complexes, which are typical densely connected within the complex and well-separated from the rest of the network. Unlike ClusterM, existing state-of-the-art algorithms (*e.g.*, NetworkBLAST, NetworkBLAST-M, and MaWISh) focus on the interaction density of the conserved protein complex but do not explicitly consider the separability of conserved complexes from the rest of the PPI network. Furthermore, compared to algorithms such as NetworkBLAST and MaWISh, which only consider proteins with homology correspondence (determined based on protein sequence similarity) across PPI networks to reduce the overall computational complexity, ClusterM does not impose such restriction and integrates the topology and homology information in a very flexible manner. Finally, another important advantage of ClusterM is that the algorithm can easily handle multiple (more than two) PPI networks and yield biologically meaningful results. While NetworkBLAST-M can also deal with multiple PPI networks, NetworkBLAST-M is not capable of handling large-scale networks.

## Conclusions

We propose a scalable algorithm—ClusterM—that explicitly characterizes the desired topolog-ical separability of protein complexes and also incorporates sequence similarity of proteins across the given PPI networks. Thanks to the computational framework used by ClusterM, it can easily handle multiple PPI networks at the same time. We have extensively compared ClusterM with other state-of-the-art algorithms on various of PPI networks. The experiments show its out-performance over other methods. In addition, ClusterM shows its potential on analyzing four PPI networks and identifying conversed protein complexes that have not been identify before.

## Materials and methods

### The ClusterM algorithm

#### Topological separability by conductance

One of the major innovations in ClusterM as well as its core strength is that it explicitly considers topological separability when searching for conserved protein complexes. In this paper, we adopt the definition of conductance to measure the topological separability of a subnetwork. Let *G*=(*V*,*E*) represent a PPI network, where *V* denotes the set of proteins in *G* and *E* is the interaction set. *A* is the corresponding adjacency matrix of *G*, where the element *A*_*ij*_=1 denotes the protein *i* interacts with the protein *j* and *A*_*ij*_=0 otherwise. The degree matrix *D* of *G* is a diagonal matrix with *D*_*ii*_=*d*_*i*_, where *d*_*i*_ is the number of interactions connecting to the protein *i*.

For a subnetwork *S* as a potential protein complex, the conductance of *S* in *G* is defined as

1 $$ \phi(S) = \frac{|E(S, \bar{S})|}{\textup{min}\left \{ vol(S), vol(\bar{S}) \right \} }, S \cup \bar{S} = V,  $$

where $E(S, \bar {S})$ denotes the set of edges between *S* and the rest of the network $\bar {S}$, and $vol(S) = \sum _{i\in S} d_{i}$ is the number of interactions in *S*. As *vol*(*S*) is typically much smaller than the total number of interactions in *G*: *vol*(*S*)≪*vol*(*V*), indicating $vol(S) = \textup {min}\left \{ vol(S), vol(\bar {S}) \right \}$, we have

2 $$ \phi(S) = \frac{|E(S, \bar{S})|}{vol(S)}=\frac{\sum_{i,j\in V_{S}} D_{ij} - A_{ij}}{\sum_{i\in V_{S}} D_{ii}},  $$

where *V*_*S*_ is the vertex set containing all vertexes in the subnetwork *S*.

#### The algorithm

ClusterM builds on the intuition that conserved protein complexes should simultaneously possess the following two properties. First, topologically, conserved protein complexes in each PPI network should be well separated from the rest of the network and proteins within the complexes should be densely connected in order to give rise to a unique and specific biological form and function. Second, across species, there should exist many homologous proteins in the conserved complexes, which can be practically reflected by high overall protein sequence similarity. Given *k* PPI networks $\mathbf {G}=\{\mathcal {G}_{1}, \mathcal {G}_{2},... \mathcal {G}_{k}\}$, where $\mathcal {G}_{j}(\mathcal {V}_{j}, \mathcal {E}_{j})$ is the *j*th network with $\mathcal {V}_{j}$ and $\mathcal {E}_{j}$ denoting the corresponding proteins and interactions respectively, we use a binary adjacency matrix *A*_*j*_ to represent $\mathcal {G}_{j}$ and use a diagonal matrix *D*_*j*_ to represent the degree matrix of $\mathcal {G}_{j}$ with the number of interactions of each protein on its diagonal.

To identify conserved protein complexes with the aforementioned properties, our ClusterM algorithm takes three major steps:

**1. Initial seeds with protein spines from multiple network alignment:** In the first step, *h* homologous seeds can be identified as protein spines $\mathcal {U} = \left \{u^{1}, u^{2},..., u^{h}\right \}$ by using a multiple network alignment method, where $u^{i} = \left \{ \left (v_{1}^{i}, v_{2}^{i},..., v_{k}^{i}\right) \mid v_{1}^{i}\in \mathcal {V}_{1}, v_{2}^{i}\in \mathcal {V}_{2},..., v_{k}^{i}\in \mathcal {V}_{k}\right \}$ constitutes the *i*th protein spine detected through network alignment. In this work, we adopt SMETANA [28], which has been shown to be accurate and scalable. When detecting these conserved protein spines, SMETANA [28] takes both protein interaction and sequence information into account. By iterating through all protein spines in $\mathcal {U}$, ClusterM adopts a divide-and-conquer strategy to identify potential conserved protein complexes from each protein spine seed across the given networks.

**2. Minimum-conductance set for topological separability (Task 1):** First, for protein $v_{j}^{i}$ in protein spine *u*^*i*^, a minimum-conductance protein set $\mathcal {\hat {H}}_{j}^{i}$ including $v_{j}^{i}$, well separated from the rest of the network $\mathcal {G}_{j}$, is identified based on a novel local optimization algorithm. Initially, we acquire a set $\mathcal {H}_{j}^{i}$ consisting of *m* proteins obtained in terms of the ranking of the personalized PageRank vector with respect to $v_{j}^{i}$ in $\mathcal {G}_{j}$ [29]. We further refine the results to yield $\mathcal {\hat {H}}_{j}^{i}$ based on the definition of the conductance (2) by solving the following optimization problem:

3 $$ \begin{aligned} \textup{min:} & \ \frac{\mathbf{x}^{T}\left(D^{\mathcal{H}_{j}^{i}}-A^{\mathcal{H}_{j}^{i}}\right)\mathbf{x}}{\mathbf{x}^{T}D^{\mathcal{H}_{j}^{i}}\mathbf{x}}\\ s.t. & \ \mathbf{x}_{v_{j}^{i}} = 1, \ \mathbf{x}_{i} \in \{0,1\}, \end{aligned}  $$

where **x** is a binary vector with **x**_*i*_=1 indicating that protein *i* is assigned into $\mathcal {\hat {H}}_{j}^{i}$ and **x**_*i*_=0 otherwise; $A^{\mathcal {H}_{j}^{i}}$ and $D^{\mathcal {H}_{j}^{i}}$ are adjacency and degree matrices of the induced subnetwork with respect to the protein set $\mathcal {H}_{j}^{i}$. The problem can be solved by transforming it into a mixed integer program (MIP) [30]. After algebraic manipulations, (3) can be transformed into the following equivalent MIP formulation:

4 $$ \begin{aligned} \textup{min:}& \ \ z\\ s.t. & \ \ z\sum_{i} \mathbf{x}_{i}d^{\mathcal{H}_{j}^{i}}_{i} - \sum_{i} \sum_{j} (D^{\mathcal{H}_{j}^{i}} - A^{\mathcal{H}_{j}^{i}})\mathbf{x}_{i}\mathbf{x}_{j} \geq 0, \\ & \ \ \mathbf{x}_{v_{j}^{i}} = 1, \mathbf{x}_{i} \in \{0,1\}, \end{aligned}  $$

where $d^{\mathcal {H}_{j}^{i}}_{i}$ is the *i*th elements on the diagonal of $D^{\mathcal {H}_{j}^{i}}$. After using standard linearization techniques [30] to linearize the terms such as *z***x**_*i*_ and **x**_*i*_**x**_*j*_, the optimization problem can be solved exactly by existing MIP solvers. We emphasize that we here obtain the exact minimum-conductance set around the selected seed, which is critical to identify potential protein complexes with high topological separability from the rest of the networks. Because ClusterM searches for the minimum-conductance set locally around each involved protein, and since the size of each protein set $\mathcal {H}_{j}^{i}$ is much smaller than the size of the entire PPI network in such a divide-and-conquer strategy, we can efficiently obtain the minimum-conductance set $\mathcal {\hat {H}}_{j}^{i}$ in $\mathcal {H}_{j}^{i}$ based on (4) [30].

Here, $\mathcal {\hat {H}}_{j}^{i}$ is the subnetwork including protein $v_{j}^{i}$ that is well separated from the other proteins in the PPI network $\mathcal {G}_{j}$. The procedure of identifying $\mathcal {\hat {H}}_{j}^{i}$ explicitly characterizes the external separability of the subnetwork $\mathcal {\hat {H}}_{j}^{i}$, which is the main advantage of ClusterM over other existing network comparative approaches [13–15, 17, 18] that allows the algorithm to effectively capture the desired topological property of conserved protein complexes.

**3. Conserved protein complexes with desired topological and homologous properties (Task 2):** The next task is to collect $\mathbf {H}^{i} = \left \{ \mathcal {\hat {H}}_{1}^{i},..., \mathcal {\hat {H}}_{k}^{i} \right \}$ for the corresponding protein spine *u*^*i*^ with $\mathbf {S}^{i} = \{ s(p,q) \mid p \in \mathcal {\hat {H}}_{m}^{i}, q \in \mathcal {\hat {H}}_{n}^{i}, \ m\ne n, \ \forall p,q,m,n \}$ denoting potential homologous correspondence within **H**^*i*^. The homologous correspondence between proteins *p* and *q* can be approximated by the protein sequence similarity *s*(*p*,*q*)∈[0,1] [31]:

5 $$ s(p, q) = \frac{blast(p, q)}{\sqrt{blast(p, p) \times blast(q, q)}},  $$

where *blast*(*p*,*q*) is the bit score of the sequence similarity obtained by the local sequence alignment tool BLAST [20]. We note that better homologous correspondence by incorporating functional annotations may further improve the performance of conserved protein complex identification. However, we do not explore such directions to avoid biased performance evaluation due to repeated usage of functional annotations in our algorithm and evaluation metrics.

To detect conserved complexes with high interaction density as well as high sequence similarity between proteins across different species, we propose to optimize the following cost function for protein spine *u*^*i*^:

6 $$ {}\begin{aligned} \mathcal{F}^{i}= -\left (\sum_{j=1}^{k}\frac{\sum\limits_{a,b\in \mathcal{\hat{H}}_{j}^{i}}{A^{\mathcal{\hat{H}}_{j}^{i}}}(a,b)\delta_{a} \delta_{b} }{\sum\limits_{c\in \mathcal{\hat{H}}_{j}^{i}} \delta_{c}}+\lambda \frac{\sum\limits_{j\ne l}\sum\limits_{a\in \mathcal{\hat{H}}_{j}^{i}}\sum\limits_{b\in\mathcal{\hat{H}}_{l}^{i}}s(a,b)\delta_{a} \delta_{b} }{\sum\limits_{j=1}^{k}\sum\limits_{c\in \mathcal{\hat{H}}_{j}^{i}} \delta_{c}} \right), \end{aligned}  $$

where the binary value $A^{\mathcal {\hat {H}}_{j}^{i}}(a,b)$ indicates whether there is an interaction between proteins *a* and *b* in the subnetwork induced by the protein set $\mathcal {\hat {H}}_{j}^{i}$, and *δ*_*a*_ is an indicator function with *δ*_*a*_=1 if protein *a* appears in the identified conserved protein complex with respect to *u*^*i*^ and *δ*_*a*_=0 otherwise. The first term is essentially the summation of the interaction density [32] of each subnetwork, which characterizes the topological cohesiveness of each subnetwork. The second term characterizes the homology correspondence represented by the summation of the protein sequence similarities across subnetworks divided by the total number of proteins in the identified conserved protein complex. The coefficient *λ* balances the contributions from network topology and protein homology information. We optimize the cost function (6) in a greedy manner by first recruiting all the proteins in **H**^*i*^, and greedily removing proteins based on their contributions to the cost function until further deletion does not reduce the cost function any more. The greedy algorithm outputs the conserved protein complex $\mathcal {C}^{i}$ with respect to the protein spine *u*^*i*^.

**Fig. 5:**
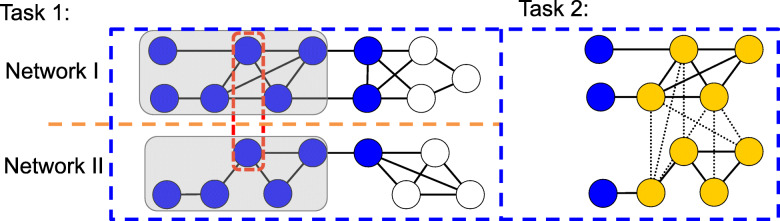
Illustration of the ClusterM procedure. The red dashed line circles the protein spine across these two networks. At first, for each network, based on the personalized PageRank vector starting from the proteins in the protein spine, we select a group of proteins marked in blue for each network. Then we refine the results by mixed integer programming and obtain the subnetworks in the gray shades, which are well separated from the rest of the networks. With them, we integrate the topological information of the selected subnetworks and the homology information between the proteins in the subnetworks, which are represented by the black dashed lines, and identify the conserved protein complex based on the cost function (6). The proteins in the golden color are proteins in the conserved protein complex identified by ClusterM

Figure 5 illustrates how ClusterM handles a protein spine in a pair of networks to search for conserved protein complex locally. Starting from the protein spine in the red dashed line, ClusterM first identifies the proteins in blue based on the personalized PageRank vectors. It further refines the results for separability by MIP (3) and recognize the proteins in the gray shade to be the subnetworks that are well separated from the rest of the corresponding networks. Finally, ClusterM finds the conserved protein complex in golden color based on the cost function (6).

In the post-processing step of ClusterM, we remove duplicated conserved protein complexes, and delete the *i*th conserved complex $\mathcal {C}^{i}$ if the summation of the sequence similarity score ($\sum _{(p,q) \in \mathcal {C}^{i}} s(p,q)$) within $\mathcal {C}^{i}$ is lower than *β* (set to *β*=0.1 in this work).

#### Scalability and parallelism

The major computation in ClusterM involves SMETANA for identifying homologous seeds, approximating the PageRank vector near a protein, solving the MIP (3), and the greedy algorithm to optimize (6). The scalability of SMETANA has been demonstrated in [28]. The computational complexity for approximating the PageRank vector near a protein is proportional to the number of involved local neighbors [29]. There is no scalability issue for solving the MIP (3) because we only consider *m* local proteins near the protein spine for individual PPI networks. Both the time and space complexities of the greedy algorithm based on (6) are *O*(*k*^2^*m*^2^), where *k* is the number of species. Therefore, due to the divide-and-conquer strategy, ClusterM has better scalability for handling multiple species.

For each protein spine *u*^*i*^, the identification of the well-separated subnetworks $\mathcal {\hat {H}}_{1}^{i},..., \mathcal {\hat {H}}_{k}^{i}$ are independent to each other. Furthermore, the protein spines and the identification of the potential conserved protein complexes are also independent. Therefore, the computation of $\mathcal {\hat {H}}_{1}^{i},..., \mathcal {\hat {H}}_{k}^{i}$ and the detection of conserved protein complexes can be easily parallelized to further reduce the total computational time for ClusterM.

### Datasets

#### Construction of DIPPPIs and IntActPPIs

We download PPI networks and protein amino acids sequences for human, yeast, fly, and worm from DIP (version 20150101) and IntAct (version 20150120). To obtain a consistent protein symbol for PPI networks and conserved protein complexes, we change all protein names utilized in this study to UniProt protein symbols. The sequence similarity for each protein pair can be obtained using a local sequence alignment tool BLAST and we select the highest BLAST bit score for each protein pair as their sequence similarity score. Then, we normalize the BLAST bit score based on the equation (5) to obtain protein homologous correspondence and threshold the sequence similarity score at 0.1.

#### The yeast-human reference conserved protein complexes

We align yeast complexes in CYC2008 [24] and human complexes in CORUM (February 2012) based on GO terms to obtain the gold standard for yeast and human conserved protein complexes. CYC2008 provides a specific GO term associated to every collected yeast complex. For human complex in CORUM, we first download the mapping of human genes and proteins to GO terms (version 20150329) [33] and annotate proteins in CORUM with GO terms that also appear in CYC2008. If a human complex contains at least half the number of proteins annotated to a GO term in CYC2008, we align the human complex and the yeast complex with the same GO term annotation.

#### High-level GO terms

When analyzing biological consistency, we only consider the high-level GO terms, which suggest specific biological functions. A GO term is defined as high-level if its information content is larger than two. The definition of the information content of a GO term *g* is *IC*=−log(|*g*|/|*root*|) [34], where “root” is the corresponding root GO term (either biological process, molecular function, or cellular component) of *g* and the operation |·| counts the number of proteins annotated to a specified GO term. Additionally, we remove GO terms “inferred from electronic annotation”, “inferred from protein interactions”, and “inferred from sequence or structural similarity”, because we utilize protein-protein interactions and the protein sequence similarities in our algorithm.

### Metrics for conserved protein complex prediction for pairwise PPI networks

The conserved protein complexes from pairwise PPI network alignment contain proteins from two species. Therefore, we need to examine the correspondence between the reference and the identified conserved complexes species by species. For all competing algorithms, we remove the identified conserved protein complexes if they contain fewer than three proteins for any species.

#### Fraction of the matched reference conserved protein complexes

For two species, given a reference conserved complex *A*={*A*_1_,*A*_2_} and an identified conserved complex *B*={*B*_1_,*B*_2_}, where *A*_1_ and *B*_1_ are complexes in the first species and *A*_2_ and *B*_2_ are complexes in the second species, we consider *B* matches *A* if the neighborhood affinity scores for complexes *w*(*A*_1_,*B*_1_)≥0.25 and *w*(*A*_2_,*B*_2_)≥0.25. The neighborhood affinity score for complexes can be calculated by

7 $$ w(X,Y) = \frac{\left | X \cap Y\right |}{\left | X \right |} \cdot \frac{\left | X \cap Y\right |}{\left | Y \right |} = \frac{\left | X \cap Y\right |^{2}}{\left | X \right |\left | Y \right |},  $$

where *X* and *Y* are two protein sets. Therefore, we can compute the fraction of the number of matched reference conserved complexes.

Assuming the reference conserved protein complexes set is *R* and the identified conserved protein complexes set is *P*, then the fraction of the number of matched reference conserved complexes can be computed as

8 $$ \textup{frac} = \frac{|C|}{|R|},  $$

where *C*={*S* | *w*(*S*_1_,*T*_1_)≥0.25 and *w*(*S*_2_,*T*_2_)≥0.25, ∀*S*∈*R*, *T*∈*P*}.

#### Accuracy score

Suppose that we have *n* reference conserved complex $A^{j} = \left \{A^{j}_{1}, A^{j}_{2}\right \}, j=1,2,...,n$, and *m* identified complex $B^{i} = \left \{ B^{i}_{1}, B^{i}_{2}\right \}, i=1,2,...,m$, and each has complexes $A^{j}_{1}$ and $B^{i}_{1}$ from the first species and another complexes $A^{j}_{2}$ and $B^{i}_{2}$ in the second species. Let *t*_*ij*_ denotes the number of proteins that exist in both the reference complex *A*^*j*^ and the identified complex *B*^*i*^, and *w*_*j*_ represent the number of proteins in the *j*th reference complex. That is, $t_{ij} = \left | A^{j}_{1} \bigcap B^{i}_{1} \right | + \left | A^{j}_{2} \bigcap B^{i}_{2} \right |$ and $w_{j} = \left | A^{j}_{1} \bigcup A^{j}_{2} \right |$. Then, complex-wise sensitivity (Sn) and positive-predictive value (PPV) can be defined as

9 $$ \textup{Sn} = \frac{\sum_{j=1}^{n} \underset{i=1,..., m}{max}t_{ij}}{\sum_{j=1}^{n} w_{j}}; \ \ \ \ \textup{PPV} = \frac{\sum_{i=1}^{m} \underset{j=1,..., n}{max}t_{ij}}{\sum_{i=1}^{m}\sum_{j=1}^{n}t_{ij}}.  $$

The geometric accuracy (Acc) score is the geometric mean of Sn and PPV: $\textup {Acc}=\sqrt {\textup {Sn} \times \textup {PPV}}$.

#### The maximum matching ratio for conserved protein complexes (MMRC)

We can quantify the overlap between the reference and the identified conserved complexes by the maximum matching ratio for conserved complexes (MMRC). MMRC is derived based on MMR [6]. The maximum matching ratio [6] is the maximum sum of weights of edges in a bipartite graph, where the two sets of nodes represent reference complexes *C* and identified complexes *S*. The bipartite graph is represented by a weighted matrix *B*_*n*×*m*_, where each weight *B*_*ij*_ is the neighborhood affinity score *w*(*c*_*i*_,*s*_*j*_) introduced earlier for the corresponding edge between complexes *c*_*i*_ and *s*_*j*_. For efficiency, we only use *B*_*ij*_≥0.25. The MMR is the solution to the following maximal matching problem.

10 $$ \begin{aligned} \textup{max:} &\ \ \frac{1}{|C|}\sum_{i=1}^{n}\sum_{j=1}^{m} B_{ij} \sigma_{c_{i}, s_{j}}\\ s.t. & \ \ \sum_{j=1}^{m} \sigma_{c_{i}, s_{j}} \leq 1 \\ & \ \ \sum_{i=1}^{n} \sigma_{c_{i}, s_{j}} \leq 1, \end{aligned}  $$

where *σ* is an indicator function with $\sigma _{c_{i}, s_{j}} = 1$ when the edge between complexes *c*_*i*_ and *s*_*j*_ is selected and $\sigma _{c_{i}, s_{j}} = 0$ otherwise.

MMRC can be obtained by modifying the overlapping weights *w*(*c*_*i*_,*s*_*j*_) between reference and identified conserved complexes. The overlapping weight between reference conserved complex *A* and identified conserved complex *B* is defined as

11 $$ o(A,B) = \frac{2w(A_{1},B_{1})\times w(A_{2},B_{2})}{w(A_{1},B_{1})+w(A_{2},B_{2})}.  $$

### Metrics for GO term consistency and coverage

We measure the functional consistency for the proteins in the conserved complexes by computing the mean normalized entropy (MNE) [7, 10] scores. We use the high-level GO term set *F* to annotate each protein in a conserved complex *R*_*i*_. The union of GO terms used for *R*_*i*_ is *F*_*i*_={*f*_1_,*f*_2_,...,*f*_*d*_}. The normalized entropy (NE) of *R*_*i*_ is computed as

12 $$ \textup{NE}(R_{i}) = \textup{NE}(p_{1}, p_{2},..., p_{d}) = -\frac{1}{\textup{log}(d)} \sum_{j} p_{j} \cdot \textup{log}\left(p_{j} \right),  $$

where *p*_*i*_ is the fraction of *R*_*i*_ with respect to the GO term *f*_*i*_. The MNE is the mean value over all NE (*R*_*i*_). For the coverage, we simply count the number of proteins in all conserved complexes, each of which consists of at least three proteins from each network.

### Parameter selection for each method

Among the tested algorithms, ClusterM and NetworkBLAST-M have only a single tuning parameter, while NetworkBLAST and MaWISh have 5 and 7 tuning parameters, respectively. In order to obtain the best performing parameters for fair comparison of the selected algorithms, we have selected the best performing parameter(s) for each algorithm by grid search. Within at most *N* combinations of *k* parameters (*p*_1_,*p*_2_,...,*p*_*k*_), we sample $n=\left \lfloor \sqrt [k]{N} \right \rfloor $ values for each parameter. The sample values are uniformly distributed between [*min*_*i*_,*max*_*i*_] for each parameter *p*_*i*_. For all the competing algorithms, we set *N*=100 and report the results with the best performing parameters.

### Implementation details

The MATLAB code and all used data are available at http://www.ece.tamu.edu/~xqian/ClusterM/. ClusterM is computationally efficient for handling multiple genome-scale PPI networks from different species. The memory consumption of ClusterM depends on the size of the subnetworks to be identified and the runtime of ClusterM can be significantly reduced by parallelization. It takes about 40 minutes for ClusterM to handle four PPI networks in IntActPPIs with 50,876 proteins in total on a laptop computer (16 GB memory and Intel i7 2.9 GHz cpu) with 2-core parallelization.

## Data Availability

The MATLAB code and all used data are available at http://www.ece.tamu.edu/~xqian/ClusterM/.

## Abbreviations

PPI: Protein-Protein Interaction
*Sce*: Saccharomyces cerevisiae
*Dme*: Drosophila melanogaster
*Cel*: Caenorhabditis elegans
*Hsa*: Homo sapiens
MMRC: The maximum matching ratio for conserved complexes
MNE: The mean normalized entropy
GO: Gene Ontology
NB: NetworkBLAST
NBM: NetworkBLAST-M
